# Systemic immune-inflammation index upon admission correlates to post-stroke cognitive impairment in patients with acute ischemic stroke

**DOI:** 10.18632/aging.205839

**Published:** 2024-05-20

**Authors:** Yongqing Cheng, Honghong Zhu, Changxia Liu, Lei Li, Fangjia Lin, Yan Guo, Cong Gu, Dingming Sun, Yang Gao, Guojun He, Shifu Sun, Shouru Xue

**Affiliations:** 1 Department of Neurology, The Yancheng Clinical College of Xuzhou Medical University, The First People’s Hospital of Yancheng, Yancheng 224000, Jiangsu, China; 2 Department of Neurology, The First Affiliated Hospital of Soochow University, Suzhou 215006, Jiangsu, China; 3 Department of Rheumatology and Immunology, The Yancheng Clinical College of Xuzhou Medical University, The First People’s Hospital of Yancheng, Yancheng 224000, Jiangsu, China

**Keywords:** post-stroke cognitive impairment, systemic immune-inflammation index, systemic inflammation response index, acute ischemic stroke, neuroinflammation

## Abstract

Background: The purpose of this prospective study was to evaluate the association of systemic immune-inflammation index (SII) and systemic inflammation response index (SIRI), with PSCI in patients with acute ischemic stroke (AIS).

Methods: First-onset AIS patients were consecutively included from January 1, 2022 to March 1, 2023. The baseline information was collected at admission. Fasting blood was drawn the next morning. Cognitive function was assessed by the Montreal Cognitive Assessment (MoCA) 3 months after onset. Logistic regression analysis was performed to explore the correlation between SII, SIRI, and PSCI. Receiver operating characteristic (ROC) was conducted to evaluate the predictive ability of SII.

Results: 332 participants were recruited, and 193 developed PSCI. Compared with patients without PSCI, the patients with PSCI had higher SII (587.75 (337.42, 988.95) vs. 345.66 (248.44, 572.89), *P*<0.001) and SIRI (1.59 (0.95, 2.84) vs. 1.02 (0.63, 1.55), *P*=0.007). SII and SIRI negatively correlated with MoCA scores (both P<0.05). The multivariable logistic regression analysis indicated that SII was independently associated with PSCI (*P*<0.001), while SIRI was not. The optimal cutoff for SII to predict PSCI was 676.83×109/L.

Conclusions: A higher level of SII upon admission was independently correlated to PSCI three months later in AIS patients.

## INTRODUCTION

According to the 2019 Global Burden of Disease (GBD) study, stroke remains the second leading cause of death worldwide [1]. As a prevalent non-motor complication of AIS, PSCI has been confirmed to be related to poor outcomes [2]. Early identification and intervention of PSCI can avoid the progressive deterioration of cognitive function and effectively improve the prognosis of patients.

Recent studies have suggested potential mechanistic links between inflammation, stroke and dementia [3, 4]. Neutrophils, lymphocytes, platelets, and monocytes are essential immune system elements [5]. The balance between innate and adaptive immunity can be better indicated by the systemic inflammation response index (SIRI) and the systemic immune-inflammation index (SII), which are calculated from the counts of neutrophils, platelets, monocytes, and lymphocytes [6]. The diagnostic and predictive efficacy of cardiovascular diseases, tumors, and inflammatory diseases has been confirmed by previous research [7–10]. Besides, another research has shown a link between the SII and a high incidence of dementia in the general public [3]. Recent findings have also confirmed the correlation between SII and hemorrhagic transformation as well as poor prognosis in AIS patients [6]. Meanwhile, the relationship between SII or SIRI and PSCI remains uncertain. Therefore, we designed this prospective cohort study to explore the association of SII and SIRI with PSCI and further evaluate their predictive value for PSCI.

## RESULTS

**Figure 1:**
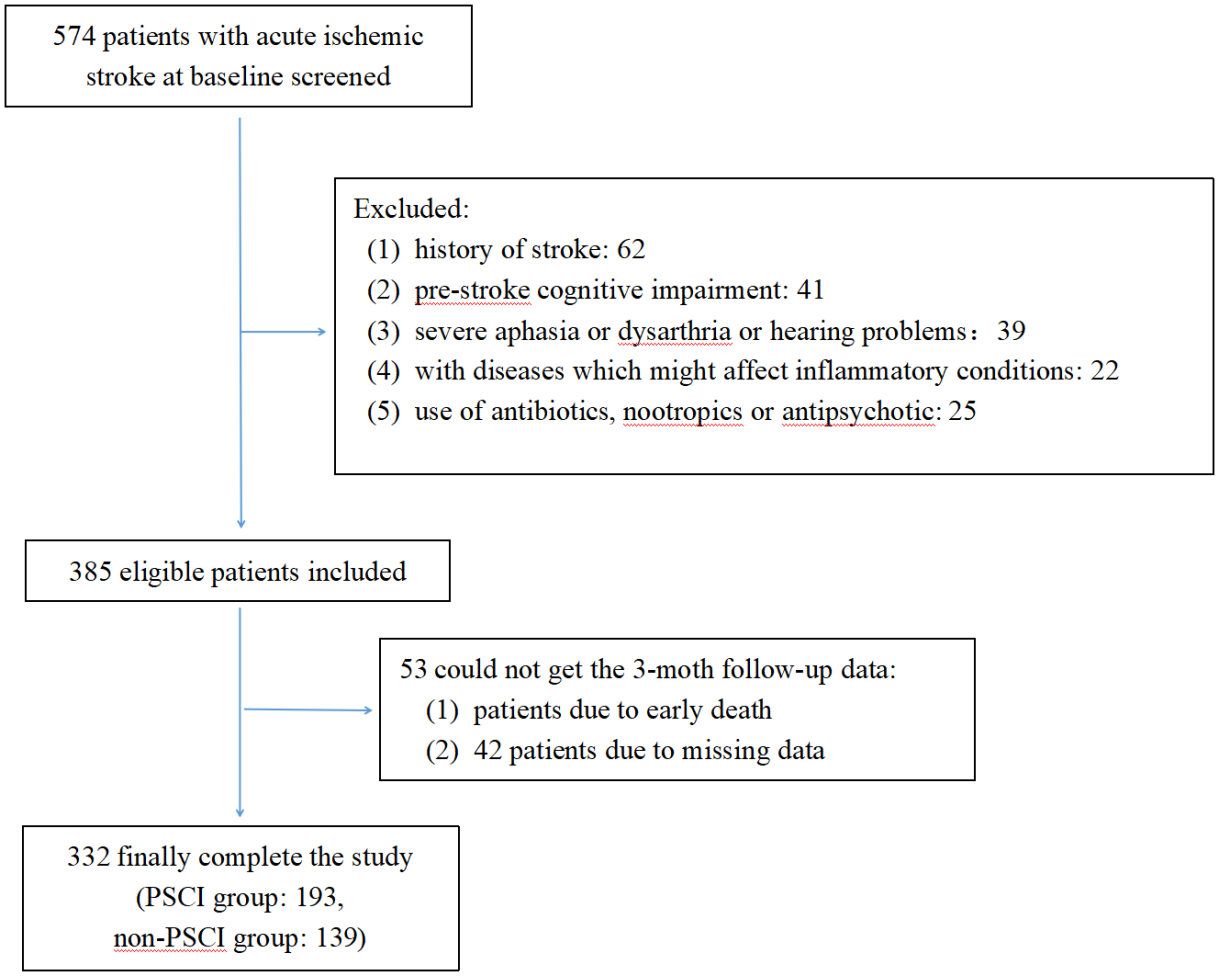
Flow diagram.

**Figure 2:**
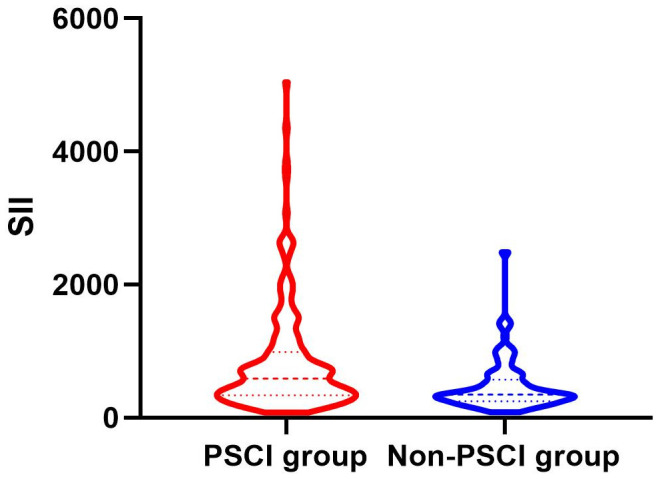
Violin plot about the distribution of SII and SIRI in the PSCI and nonPSCI subgroups.

**Figure 3:**
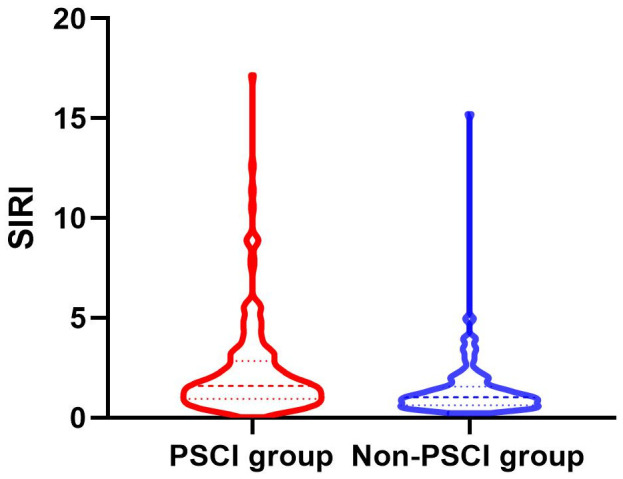
Violin plot about the distribution of SII and SIRI in the PSCI and nonPSCI subgroups.

Between January 2022 and March 2023, a consecutive screening of 574 patients with AIS was performed. We excluded 242 patients on the basis of exclusion criteria and incomplete follow-up data. Finally, the study enrolled 332 participants, 203 of whom were male (Figure 1). The mean age of the participants was 68 years old, with an interquartile range of 58-76, and 58.13% of them developed PSCI three months later. The tertile levels of SII and SIRI were as follows: Tertile 1 (76.67≤SII<335.02), Tertile 2 (335.02≤SII<683.03), Tertile 3 (683.03≤SII≤5048.81) and Tertile 1 (0.02≤SIRI<0.95), Tertile 2 (0.95≤SIRI<1.79), Tertile 3 (1.79≤SIRI≤17.17). Violin plots about the distribution of SII and SIRI in subgroups are shown in Figures 2, 3.

### Correlation between SII, SIRI, and MoCA score

**Figure 4:**
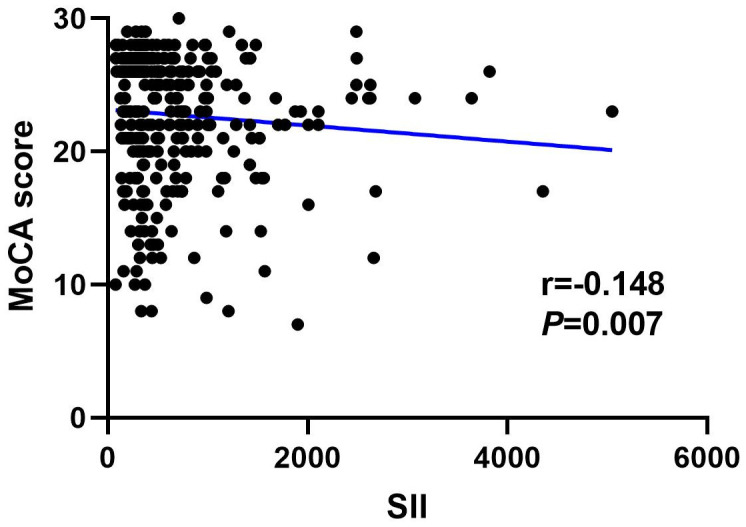
Spearman correlation analysis between SII, SIRI and MoCA score.

**Figure 5:**
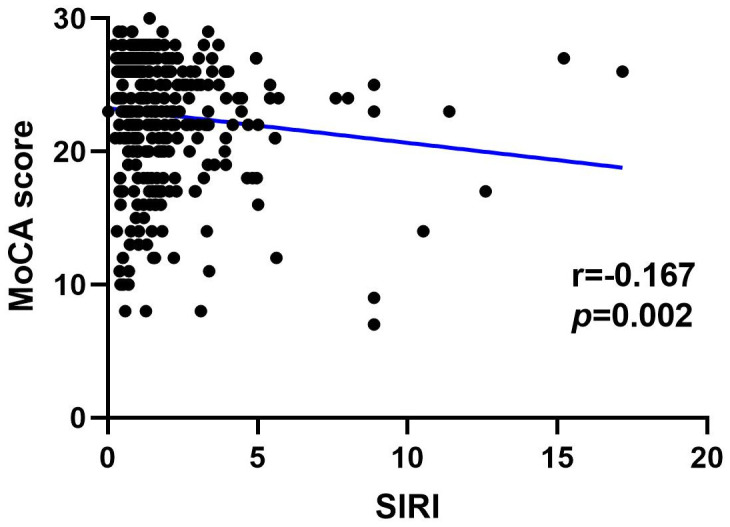
Spearman correlation analysis between SII, SIRI and MoCA score.

Figures 4, 5 show the results of Spearman’s analysis of the correlation between SII and MoCA and SIRI and MoCA scores, respectively. The negative correlations of SII and SIRI with MoCA scores were clarified in the results (r=-0.143, *P*=0.007 and r=-0.167, *P*=0.002, respectively).

### Contrasts in characteristics among the PSCI and non-PSCI subgroups

The cognitive function of participants was assessed using the MoCA scale three months after the onset, resulting in the diagnosis of PSCI in 193 patients. The participants were segregated into subgroups based on their cognitive function. Table 1 presents the differences in characteristics between the PSCI and non-PSCI subgroups. The results indicated that patients with PSCI had higher levels of age, NIHSS, cerebral infarct volume, Fazekas score, FPG, TG, Neutrophils, monocyte, SII, SIRI (all *P*<0.005), as well as lower levels of education, HDL, lymphocytes, MoCA score (all *P*<0.005). Moreover, patients with PSCI also had higher proportions of atrial fibrillation (*P*=0.027), drinking (*P*=0.008), and cortical infarction (*P*=0.003).

**Table 1:** Characteristics between PSCI group and non-PSCI group.

**Baseline characteristics**	**PSCI (n=193)**	**Non-PSCI (n=139)**	***P***
**Demographics**			
Male, n (%)	115 (59.6)	88 (63.3)	0.496
Age, median (IQR) (years)	72.0 (64.0, 79.0)	61.0 (52.0, 69.0)	<0.001
BMI, median (IQR) (Kg/m^2^)	24.49 (22.29, 27.02)	24.77 (22.49, 26.93)	0.465
Education level, n (%)			<0.001
Illiterate	50 (25.9)	7 (5.0)	
Primary school	78 (40.4)	50 (36.0)	
Secondary school or above	65 (33.7)	82 (59.0)	
**Medical history, n (%)**			
Hypertension	131 (67.9)	80 (57.6)	0.064
Diabetes mellitus	64 (33.2)	38 (27.3)	0.279
Coronary artery disease	27 (14.0)	20 (14.4)	0.920
Atrial fibrillation	35 (18.1)	13 (9.4)	0.027
Smoking	73 (37.8)	50 (36.0)	0.818
Drinking	81 (42.0)	38 (27.3)	0.008
**Clinical characteristics**			
NIHSS on admission, median (IQR)	6.0 (2.0, 10.5)	3.0 (2.0, 6.0)	<0.001
Cerebral infarct volume, median (IQR) (cm^3^)	2.3 (0.62, 6.50)	0.94 (0.40, 2.30)	<0.001
Cortical infarction, n (%)	96 (49.7)	46 (33.1)	0.003
carotid plaque, n (%)	171 (88.6)	118 (84.9)	0.408
Carotid artery stenosis, n (%)	58 (30.1)	44 (31.7)	0.81
Fazekas score, median (IQR)	4.0 (3.0, 5.0)	3.0 (3.0, 4.0)	<0.001
Stroke etiology, n (%)			0.055
LAA	113 (58.5)	79 (56.8)	
Cardioembolism	25 (13.0)	7 (5.0)	
Small-vessel occlusion	48 (24.9)	46 (33.1)	
Undetermined/unclassified	7 (3.6)	7 (5.0)	
**Laboratory characteristics**			
Hcy, median (IQR) (μmol/L)	11.40 (8.75, 16.15)	11.20 (8.70, 16.80)	0.887
FBG, median (IQR) (mmol/L)	5.81 (5.08, 8.35)	5.17 (4.63, 6.32)	<0.001
Uric acid, median (IQR) (μmol/L)	325.00 (258.60, 406.15)	321.3 (268.10, 382.00)	0.58
HbA1c, median (IQR) (%)	5.7 (5.4, 6.7)	5.7 (5.3, 6.5)	0.167
TG, median (IQR) (mg/dL)	1.55 (0.98, 2.28)	1.37 (1.07, 1.72)	0.03
TC, median (IQR) (mmol/L)	4.46 (3.68, 5.41)	4.37 (3.74, 5.25)	0.608
HDL-C, median (IQR) (mmol/L)	1.00 (0.86, 1.19)	1.09 (0.98, 1.24)	0.001
LDL-C, median (IQR) (mmol/L)	2.70 (2.17, 3.34)	2.63 (2.13, 3.35)	0.508
Leukocyte, median (IQR) (10^9^/L)	7.04 (5.40, 8.96)	6.07 (4.98, 7.52)	0.001
Neutrophils, median (IQR) (10^9^/L)	4.49 (3.20, 6.77)	3.61 (2.78, 4.62)	<0.001
Lymphocytes, median (IQR) (10^9^/L)	1.47 (1.11, 1.92)	1.59 (1.30, 2.01)	0.031
Monocyte, median (IQR) (10^9^/L)	0.53 (0.42, 0.67)	0.48 (0.37, 0.59)	0.007
Platelets, median (IQR) (10^9^/L)	184.0 (144.5, 241.5)	184.0 (136.0, 216.0)	0.136
SII, median (IQR) (10^9^/L)	587.75 (337.42, 988.95)	345.66 (248.44, 572.89)	<0.001
SIRI, median (IQR) (10^9^/L)	1.59 (0.95, 2.84)	1.02 (0.63, 1.55)	<0.001
SII tertiles, n (%)			<0.001
T1	47 (24.4)	64 (46.0)	
T2	61 (31.6)	50 (36.0)	
T3	85 (44.0)	25 (18.0)	
SIRI tertiles, n (%)			<0.001
T1	48 (24.9)	63 (45.3)	
T2	64 (33.2)	47 (33.8)	
T3	81 (42.0)	29 (20.9)	
MoCA, median (IQR)	21 (17, 23)	27 (26, 27)	<0.001

Abbreviations: PSCI, post-stroke cognitive impairment; BMI, body mass index; NIHSS, National Institutes of Health Stroke Scale; LAA, large artery atherosclerosis; Hcy, homocysteine; FPG, fasting plasma glucose; HbA1c, glycosylated hemoglobin A1c; TG, triglyceride; TC, total cholesterol; HDL-C, high-density lipoprotein cholesterol; LDL-C, low-density lipoprotein cholesterol; SII, systemic immune-inflammation index; SIRI, systemic inflammation response index; MoCA, Montreal Cognitive Assessment.

### Relationship between SII, SIRI and PSCI

The findings from the logistic regression models with SII and SIRI as continuous variables are presented in Table 2. Results from the univariable logistic regression analysis demonstrated a significant association of PSCI with age, education level, history of atrial fibrillation, history of drinking, NIHSS, infarction volume, cortical infarction, Fazekas score and some laboratory data (including FPG, leukocyte, neutrophils, monocyte, SII, SIRI, TG, and HDL-C) (all *P*<0.05). After controlling for variables with *P*<0.1 in the univariable regression analysis, education level (OR=0.258, *P*=0.011), age (OR=1.089, *P*<0.001), history of drinking (OR=2.035, *P*=0.026), NIHSS (OR=1.176, *P*<0.001), cerebral infarct volume (OR=1.068, *P*=0.016), cortical infarction (OR=1.064, *P*=0.034), FPG (OR=1.231, *P*=0.001), TG (OR=2.193, *P*<0.001) and SII (OR=1.002, *P*<0.001) were proved to be independently associated with PSCI in the multivariable regression analysis. Furthermore, SII and SIRI were then entered into the multivariable regression model as tertiles. The findings demonstrated that when the first tertile was taken as a reference, the second and third tertile of SII were both independent risk factors for PSCI (OR=2.355, P=0.021 and OR=10.369, *P*<0.001, respectively). However, no significant correlation between SIRI and PSCI was found (Table 3).

**Table 2:** Univariable and multivariable analyses for the potential risk factors associated with PSCI, including SII and SIRI as continuous variables by logistic regression.

**Baseline characteristics**	**Univariable analysis**		**Multivariable analysis**	
	**OR (95%CI)**	***P*-value**	**Adjusted OR (95%CI)**	***P*-value**
**Demographics**				
Male	0.854 (0.545-1.339)	0.492		
Age	1.072 (1.050-1.095)	<0.001	1.089 (1.059-1.119)	<0.001
BMI	0.960 (0.896-1.028)	0.244		
Education level				
Illiterate	reference		reference	
Primary school	0.218 (0.092-0.520)	0.001	0.422 (0.149-1.199)	0.105
Secondary school or above	0.111 (0.047-0.261)	<0.001	0.258 (0.090-0.737)	0.011
**Medical history**				
Hypertension	1.558 (0.991-2.449)	0.054		
Diabetes mellitus	1.319 (0.817-2.128)	0.257		
Coronary artery disease	0.968 (0.518-1.807)	0.918		
Atrial fibrillation	2.147 (1.090-4.231)	0.027	-	0.962
Smoking	1.083 (0.689-1.702)	0.730		
Drinking	1.922 (1.201-3.075)	0.006	2.035 (1.089-3.803)	0.026
**Clinical characteristics**				
NIHSS on admission	1.171 (1.101-1.246)	<0.001	1.176 (1.083-1.278)	<0.001
Cerebral infarct volume	1.077 (1.031-1.125)	0.001	1.068 (1.012-1.126)	0.016
Cortical infarction	2.001 (1.273-3.145)	0.003	1.964 (1.051-3.670)	0.034
Carotid atherosclerosis	1.383 (0.728-2.629)	0.322		
Carotid artery stenosis	0.928 (0.579-1.487)	0.755		
Fazekas score	1.734 (1.382-2.176)	<0.001	-	0.525
Stroke etiology				
LAA	Reference		Reference	
Cardioembolism	2.497 (1.029-6.056)	0.043	-	0.602
Small-vessel occlusion	0.730 (0.444-1.198)	0.213	-	0.315
Undetermined/unclassified	0.699 (0.236-2.072)	0.518	-	0.873
**Laboratory characteristics**				
Hcy	1.008 (0.967-1.051)	0.704		
FPG	1.177 (1.073-1.290)	0.001	1.231 (1.090-1.391)	0.001
Uric acid	1.001 (0.999-1.003)	0.369		
HbA1c	1.068 (0.926-1.232)	0.366		
TG	1.462 (1.120-1.910)	0.005	2.193 (1.471-3.270)	<0.001
TC	1.098 (0.922-1.307)	0.295		
HDL-C	0.395 (0.201-0.773)	0.007	-	0.066
LDL-C	1.126 (0.880-1.441)	0.346		
Leukocyte	1.181 (1.071-1.302)	0.001	-	0.913
Neutrophils	1.268 (1.131-1.421)	<0.001	-	0.828
Lymphocytes	0.718 (0.510-1.011)	0.058	-	0.856
Monocyte	4.776 (1.539-14.818)	0.007	-	0.599
Platelets	1.003 (1.000-1.006)	0.076	-	0.989
SII	1.001 (1.001-1.002)	<0.001	1.002 (1.001-1.002)	<0.001
SIRI	1.429 (1.189-1,719)	<0.001	-	0.728

Abbreviations: PSCI, post-stroke cognitive impairment; IQR, interquartile range; BMI, body mass index; NIHSS, National Institutes of Health Stroke Scale; LAA, large artery atherosclerosis; Hcy, homocysteine; FPG, fasting plasma glucose; HbA1c, glycosylated hemoglobin A1c; TG, triglyceride; TC, total cholesterol; HDL-C, high-density lipoprotein cholesterol; LDL-C, low-density lipoprotein cholesterol; SII, systemic immune-inflammation index; SIRI, systemic inflammation response index; MoCA, Montreal Cognitive Assessment.

**Table 3:** Multivariable analyses for the potential risk factors associated with PSCI, including SII and SIRI as tertiles by logistic regression.

**Baseline characteristics**	**Multivariable analysis**	
	**Adjusted OR (95%CI)**	***P-value***
**Demographics**		
Age	1.090 (1.060-1.122)	<0.001
Education level		
Illiterate	reference	
Primary school	0.448 (0.153-1.313)	0.143
Secondary school or above	0.264 (0.089-0.780)	0.016
**Medical history**		
Hypertension	-	0.990
Atrial fibrillation	-	0.880
Drinking	2.060 (1.093-3.884)	0.025
**Clinical characteristics**		
NIHSS on admission	1.188 (1.091-1.295)	<0.001
Cerebral infarct volume	1.072 (1.014-1.133)	0.014
Cortical infarction	1.935 (1.016-3.687)	0.045
Fazekas score		0.386
Stroke etiology		
LAA	Reference	
Cardioembolism	-	0.537
Small-vessel occlusion	-	0.160
Undetermined/unclassified	-	0.927
**Laboratory characteristics**		
FPG	1.255 (1.104-1.427)	0.001
TG	2.213 (1.451-3.375)	<0.001
HDL-C	0.371 (0.137-1.000)	0.050
Leukocyte	-	0.945
Neutrophils	-	0.991
Lymphocytes	-	0.870
Monocyte	-	0.819
Platelets	-	0.948
SII tertiles		
T1	reference	
T2	2.355 (1.138-4.877)	0.021
T3	10.369 (4.460-24.107)	<0.001
SIRI tertiles		
T1	reference	
T2	-	0.822
T3	-	0.572

Abbreviations: PSCI, post-stroke cognitive impairment; BMI, body mass index; NIHSS, National Institutes of Health Stroke Scale; LAA, large artery atherosclerosis; FPG, fasting plasma glucose; TG, triglyceride; HDL-C, high-density lipoprotein cholesterol; SII, systemic immune-inflammation index; SIRI, systemic inflammation response index; MoCA, Montreal Cognitive Assessment.

### ROC analysis of SII for predicting PSCI

**Figure 6:**
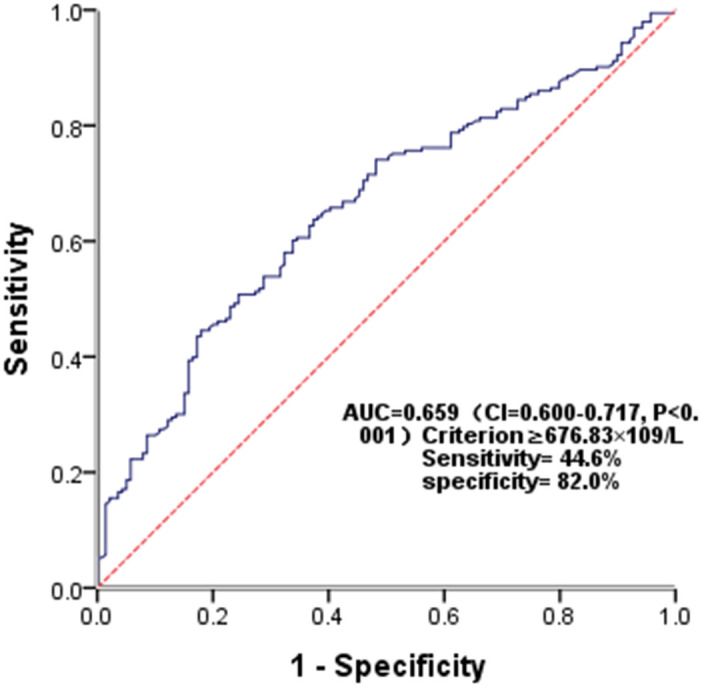
Receiver operating characteristic (ROC) curve for SII as a predictor of PSCI.

The diagnostic utility of SII in predicting PSCI was assessed using ROC analysis, with the AUC of 0.659 (P<0.001). (Figure 6). The optimal cutoff value was ≥676.83×10^9^/L, and the sensitivity and specificity were 44.6% and 82.0%, respectively.

## DISCUSSION

The inflammation response has been reported to be crucial in stroke and PSCI pathobiology [11, 12]. Previous studies have revealed that systemic inflammation processes are closely related to endothelial dysfunction, cell death, blood-brain barrier (BBB) disruption, cerebral blood flow autoregulation disorder and platelet aggregation [5, 13]. Studies consecutively showed that PSCI was associated with some inflammatory biomarkers and cytokines [14, 15]. Otherwise, as important primary immune mediators that can release inflammatory signals, infiltrating leukocytes, including neutrophils, monocytes, and lymphocytes, have been reported to be related to stroke and dementia [16, 17]. Previous studies have shown that peripheral neutrophils and neutrophil to lymphocyte ratio (NLR) correlate to poor prognosis and hemorrhagic transformation of ischemic stroke, as well as cerebral small vessel disease and dementia [18–20].

The SII and SIRI derived from different blood cells can better reflect the inflammation or immune status than one cell alone. Hu et al. created SII and reported that SII was an effective predictor of poor outcomes of patients after an operation for hepatocellular carcinoma [9]. Meanwhile, SII was also considered to be correlated to unfavorable outcomes of various tumors such as cholangiocarcinoma, lung cancer, gliomas, etc. [10, 21, 22]. Subsequently, there has been an established link between SII and the occurrence and prognosis of chronic heart failure and coronary heart disease [23, 24]. A recent system review showed that elevated SII could significantly increase the risk of vascular disease, including ischemic stroke, hemorrhagic stroke, myocardial infarction, and peripheral arterial disease [7]. According to a recent large-scale general population study, elevated SII and SIRI could increase the incidence of stroke and all-cause death [25]. In addition, SII on admission was reported to be positively associated with symptomatic intracranial hemorrhage after endovascular treatment in AIS patients with large vessel occlusion [24]. Recently, several research have confirmed the potential relationship between SII and cognitive impairment. According to a retrospective study, elevated SII was closely related to the occurrence of postoperative cognitive decline [26]. Another research also showed a strong correlation between SII and cerebral small vessel disease (CSVD) and cognitive impairment [27]. Therefore, we speculate that SII and PSCI might have a potential relationship. However, there are few reports on the correlation between them. In this research, we discovered that SII was independently correlated to PSCI and might be used as a valid predictor.

The specific mechanisms for the association between SII and PSCI are not yet well understood. Nevertheless, it is hypothesized that blood-brain barrier disruption, endothelial dysfunction, CVSD, and neuroinflammation could have significant implications [11, 28]. Many studies have shown that neutrophils, platelets, and lymphocytes, essential components of SII, were related to endothelial dysfunction and blood-brain barrier disruption [17, 7]. Neutrophils play a negative role by releasing reactive oxides, synthesizing cytokines, intercellular adhesion molecules, and other inflammatory mediators, while platelets secrete pro-inflammatory cytokines and growth factors. [29–31]. Furthermore, accumulating evidence has shown that increased SII levels are related to more severe CSVD, which plays a crucial role in cognitive impairment [32]. A community-based population study has demonstrated that individuals with a higher SII had an increased risk of moderate-to-severe enlarged perivascular space (EPVS) and modified white matter hyperintensity (WMH) burden [19]. In addition, peripheral inflammation can penetrate the BBB and induce central neuroinflammation, ultimately contributing causally to cognitive impairment [33]. Emerging evidence has suggested that neuroinflammation plays an active role rather than being passive activation in the pathogenesis of cognitive impairment [13, 34].

Our study confirmed the potential association of SII with PSCI. Nevertheless, some limitations should be noted. First, a few patients with severe aphasia, dysarthria or disturbance of consciousness were not included, which could lead to bias. Second, although we excluded patients with previous stroke, it is difficult to guarantee that the cognitive impairment was exclusively stroke-related because we could not accurately assess pre-stroke cognitive function. Third, we only measured SII and SIRI levels at admission and MoCA scores three months after stroke. The lack of serial detections for levels of SII, SIRI, and cognitive performance may have obscured any potential impact of treatment interventions on the noted correlation. Fourth, we could not rule out the possible impact of some potential risk factors we did not measure, such as ApoE status. Finally, the single center and restricted sample size limit the generalization of the results of our study, and the predictive effect of SII for PSCI in this study was statistically significant but not strong enough.

## CONCLUSIONS

The potential correlation between SII and PSCI was confirmed by our study. A high level of SII at admission might be an effective predictor of PSCI. Further exploration of the potential mechanism might provide new targets for PSCI treatment.

## MATERIALS AND METHODS

### Patient enrollment

This was a prospective cohort study conducted in the First People’s Hospital of Yancheng. The participants were consecutively screened from inpatient department between January 1, 2022, and March 1, 2023. Patients were enrolled if they met the following criteria: (1) ≥ 18 years old, (2) met the World Health Organization diagnostic criteria for AIS confirmed by neuroimaging, (3) admission within seven days of symptom onset, (4) first stroke. Exclusion criteria were as follows: (1) with pre-existing cognitive disorder from diverse diseases, (2) with neurological dysfunctions that may affect cognitive evaluation, such as hearing impairment, aphasia or dysarthria, (3) with diseases which might affect inflammatory conditions, such as blood disease, acute infection, malignant tumors, or trauma, (4) intake of antibiotics, psychotropic or nootropics medications within three months.

### Baseline clinical and laboratory data

Clinical and laboratory data were collected in a manner similar to that described in our other article [35]. On admission, we used a standard questionnaire to collect clinical data and to assess the presence of pre-existing cognitive disorder. Baseline demographics (gender, age, education, body mass index (BMI), history of smoking and drinking, as well as medical history (diabetes, hypertension, atrial fibrillation, and coronary artery disease) were collected. We classified the patients into three education levels: those with less than one year of education were classified as illiterate, those with one to six years of education were classified as primary school, and those with more than six years were classified as secondary school or above. The etiology and severity of stroke were determined according to the Trial of Org 10172 in Acute Stroke Treatment (TOAST) criteria and National Institutes of Health Stroke Scale (NIHSS), respectively. Fasting blood samples were obtained from all patients the morning after admission and processed and recorded by a single laboratory physician. The laboratory data included glycosylated hemoglobin A1 (HbA1c), fasting plasma glucose (FPG), uric acid (UA), peripheral blood cell counts, triglyceride (TG), total cholesterol (TC), low-density lipoprotein (LDL), high-density lipoprotein (HDL), and homocysteine (Hcy). We calculated SII versus SIRI using the following formula: platelet count × neutrophil count/lymphocyte count, neutrophil count × monocyte count / lymphocyte count, respectively [9, 25]. All blood laboratory assessments were conducted in the hospital’s clinical laboratory. An auto-analyzer (XN-1000, Sysmex, Kobe, Japan) analyzed all blood cell counts, and other profiles were assessed with an automated biochemical analyzer.

All participants underwent brain MRI scans within 72 hours after admission, and imaging data were collected and analyzed by a doctor from the imaging department. The severity of white matter hyperintensities was assessed using the Fezakas scores, which ranges from 0 to 6. The infarct volume was calculated by multiplying the infarct area of each slice by the slice thickness on the DWI sequence and then summing [36]. The carotid plaque and stenosis were evaluated by carotid ultrasound or CTA.

### Assessments of cognitive function

Cognitive function was assessed at 3 months after stroke by two trained neurologists using the MoCA scale. The total score was 0-30 points, and a score < 26 points was defined as PSCI [37]. One point was added to the total score if the patient had less than 12 years of education as MoCA is closely associated with educational level.

### Statistical analysis

Statistical analyses were conducted by SPSS version 23.0 (IBM, New York, NY, USA) and GraphPad Prism version 8.0.2 (GraphPad Software, San Diego, CA, USA). Continuous variables were presented as the mean±standard deviation or median (interquartile range [IQR]) and categorical variables were presented as numbers (percentages [%]). We compared all characteristics between the PSCI and nonPSCI subgroups, as well as among the SII and SIRI tertiles. The Chi-square test or Fisher’s exact test was used for categorical variables (such as sex and medical history), and one-way ANOVA, analysis of variance, the Mann-Whitney U test or Kruskal-Wallis test was used for continuous variables (such as age). The association between SII, SIRI and MoCA score was analyzed by Spearman’s correlation. Univariable binary regression analysis was conducted to investigate the association of baseline characteristics with PSCI, and all variables with *P* < 0.1 were entered into the subsequent multivariable logistic regression model. Odds ratio (OR) or adjusted OR combined with 95% confidence intervals (CIs) demonstrate associations. Subsequently, we evaluated the potential predictive effect of SII on PSCI using ROC curve. All statistical analyses were defined as statistically significant with a two-sided *P* < 0.05.

## References

[1] GBD 2019 Stroke Collaborators. Global, regional, and national burden of stroke and its risk factors, 1990-2019: a systematic analysis for the Global Burden of Disease Study 2019. Lancet Neurol. 2021; 20:795–820. 10.1016/S1474-4422(21)00252-0 34487721 PMC8443449

[2] Lo JW, Crawford JD, Desmond DW, Godefroy O, Jokinen H, Mahinrad S, Bae HJ, Lim JS, Köhler S, Douven E, Staals J, Chen C, Xu X, et al, and Stroke and Cognition (STROKOG) Collaboration. Profile of and risk factors for poststroke cognitive impairment in diverse ethnoregional groups. Neurology. 2019; 93:e2257–71. 10.1212/WNL.0000000000008612 31712368 PMC6937495

[3] van der Willik KD, Fani L, Rizopoulos D, Licher S, Fest J, Schagen SB, Ikram MK, Ikram MA. Balance between innate versus adaptive immune system and the risk of dementia: a population-based cohort study. J Neuroinflammation. 2019; 16:68. 10.1186/s12974-019-1454-z 30927918 PMC6441146

[4] Anrather J, Iadecola C. Inflammation and Stroke: An Overview. Neurotherapeutics. 2016; 13:661–70. 10.1007/s13311-016-0483-x 27730544 PMC5081118

[5] Elkind MS. Inflammatory mechanisms of stroke. Stroke. 2010; 41:S3–8. 10.1161/STROKEAHA.110.594945 20876499 PMC2963080

[6] Huang YW, Yin XS, Li ZP. Association of the systemic immune-inflammation index (SII) and clinical outcomes in patients with stroke: A systematic review and meta-analysis. Front Immunol. 2022; 13:1090305. 10.3389/fimmu.2022.1090305 36591305 PMC9797819

[7] Ye Z, Hu T, Wang J, Xiao R, Liao X, Liu M, Sun Z. Systemic immune-inflammation index as a potential biomarker of cardiovascular diseases: A systematic review and meta-analysis. Front Cardiovasc Med. 2022; 9:933913. 10.3389/fcvm.2022.933913 36003917 PMC9393310

[8] Shi S, Kong S, Ni W, Lu Y, Li J, Huang Y, Chen J, Lin K, Li Y, Ke J, Zhou H. Association of the Systemic Immune-Inflammation Index with Outcomes in Acute Coronary Syndrome Patients with Chronic Kidney Disease. J Inflamm Res. 2023; 16:1343–56. 10.2147/JIR.S397615 37006811 PMC10065009

[9] Hu B, Yang XR, Xu Y, Sun YF, Sun C, Guo W, Zhang X, Wang WM, Qiu SJ, Zhou J, Fan J. Systemic immune-inflammation index predicts prognosis of patients after curative resection for hepatocellular carcinoma. Clin Cancer Res. 2014; 20:6212–22. 10.1158/1078-0432.CCR-14-0442 25271081

[10] Mazzella A, Maiolino E, Maisonneuve P, Loi M, Alifano M. Systemic Inflammation and Lung Cancer: Is It a Real Paradigm? Prognostic Value of Inflammatory Indexes in Patients with Resected Non-Small-Cell Lung Cancer. Cancers (Basel). 2023; 15:1854. 10.3390/cancers15061854 36980740 PMC10046843

[11] Pendlebury ST, Rothwell PM. Prevalence, incidence, and factors associated with pre-stroke and post-stroke dementia: a systematic review and meta-analysis. Lancet Neurol. 2009; 8:1006–18. 10.1016/S1474-4422(09)70236-4 19782001

[12] Becker KJ, Buckwalter M. Stroke, Inflammation and the Immune Response: Dawn of a New Era. Neurotherapeutics. 2016; 13:659–60. 10.1007/s13311-016-0478-7 27677606 PMC5081111

[13] Heneka MT, Carson MJ, El Khoury J, Landreth GE, Brosseron F, Feinstein DL, Jacobs AH, Wyss-Coray T, Vitorica J, Ransohoff RM, Herrup K, Frautschy SA, Finsen B, et al. Neuroinflammation in Alzheimer’s disease. Lancet Neurol. 2015; 14:388–405. 10.1016/S1474-4422(15)70016-5 25792098 PMC5909703

[14] Huang LC, Hsieh SW, Tsai CC, Chen CH, Yang YH. The Role of Cilostazol and Inflammation in Cognitive Impairment After Ischemic Stroke. Am J Alzheimers Dis Other Demen. 2021; 36:15333175211016185. 10.1177/15333175211016185 34008421 PMC10624089

[15] Cui L, Lu P, Li S, Pan Y, Wang M, Li Z, Liao X, Wang Y. Relationship Among Homocysteine, Inflammation and Cognitive Impairment in Patients with Acute Ischemic Stroke and Transient Ischemic Attack. Neuropsychiatr Dis Treat. 2021; 17:3607–16. 10.2147/NDT.S333753 34924754 PMC8674150

[16] Ao LY, Yan YY, Zhou L, Li CY, Li WT, Fang WR, Li YM. Immune Cells After Ischemic Stroke Onset: Roles, Migration, and Target Intervention. J Mol Neurosci. 2018; 66:342–55. 10.1007/s12031-018-1173-4 30276612

[17] Kim JY, Park J, Chang JY, Kim SH, Lee JE. Inflammation after Ischemic Stroke: The Role of Leukocytes and Glial Cells. Exp Neurobiol. 2016; 25:241–51. 10.5607/en.2016.25.5.241 27790058 PMC5081470

[18] Jiang L, Cai X, Yao D, Jing J, Mei L, Yang Y, Li S, Jin A, Meng X, Li H, Wei T, Wang Y, Pan Y, Wang Y. Association of inflammatory markers with cerebral small vessel disease in community-based population. J Neuroinflammation. 2022; 19:106. 10.1186/s12974-022-02468-0 35513834 PMC9072153

[19] Maestrini I, Strbian D, Gautier S, Haapaniemi E, Moulin S, Sairanen T, Dequatre-Ponchelle N, Sibolt G, Cordonnier C, Melkas S, Leys D, Tatlisumak T, Bordet R. Higher neutrophil counts before thrombolysis for cerebral ischemia predict worse outcomes. Neurology. 2015; 85:1408–16. 10.1212/WNL.0000000000002029 26362283 PMC4626239

[20] Zhang R, Jin F, Zheng L, Liao T, Guan G, Wang J, Zhao S, Fei S, Chu Z, Xu Y. Neutrophil to High-Density Lipoprotein Ratio is Associated with Hemorrhagic Transformation in Patients with Acute Ischemic Stroke. J Inflamm Res. 2022; 15:6073–85. 10.2147/JIR.S381036 36386588 PMC9642365

[21] Zhang S, Ni Q. Prognostic role of the pretreatment systemic immune-inflammation index in patients with glioma: A meta-analysis. Front Neurol. 2023; 14:1094364. 10.3389/fneur.2023.1094364 36970508 PMC10030933

[22] Bailey-Whyte M, Minas TZ, Dorsey TH, Smith CJ, Loffredo CA, Ambs S. Systemic Inflammation Indices and Association with Prostate Cancer Survival in a Diverse Patient Cohort. Cancers (Basel). 2023; 15:1869. 10.3390/cancers15061869 36980755 PMC10047449

[23] Yang YL, Wu CH, Hsu PF, Chen SC, Huang SS, Chan WL, Lin SJ, Chou CY, Chen JW, Pan JP, Charng MJ, Chen YH, Wu TC, et al. Systemic immune-inflammation index (SII) predicted clinical outcome in patients with coronary artery disease. Eur J Clin Invest. 2020; 50:e13230. 10.1111/eci.13230 32291748

[24] Yang Y, Cui T, Bai X, Wang A, Zhang X, Wan J, Wang C, Lu K, Hu F, Wu B. Association Between Systemic Immune-Inflammation Index and Symptomatic Intracranial Hemorrhage in Acute Ischemic Stroke Patients Undergoing Endovascular Treatment. Curr Neurovasc Res. 2022; 19:83–91. 10.2174/1567202619666220406102429 35388755

[25] Jin Z, Wu Q, Chen S, Gao J, Li X, Zhang X, Zhou Y, He D, Cheng Z, Zhu Y, Wu S. The Associations of Two Novel Inflammation Indexes, SII and SIRI with the Risks for Cardiovascular Diseases and All-Cause Mortality: A Ten-Year Follow-Up Study in 85,154 Individuals. J Inflamm Res. 2021; 14:131–40. 10.2147/JIR.S283835 33500649 PMC7822090

[26] Lu W, Zhang K, Chang X, Yu X, Bian J. The Association Between Systemic Immune-Inflammation Index and Postoperative Cognitive Decline in Elderly Patients. Clin Interv Aging. 2022; 17:699–705. 10.2147/CIA.S357319 35535363 PMC9078355

[27] Xiao Y, Teng Z, Xu J, Qi Q, Guan T, Jiang X, Chen H, Xie X, Dong Y, Lv P. Systemic Immune-Inflammation Index is Associated with Cerebral Small Vessel Disease Burden and Cognitive Impairment. Neuropsychiatr Dis Treat. 2023; 19:403–13. 10.2147/NDT.S401098 36852257 PMC9960781

[28] Thiel A, Cechetto DF, Heiss WD, Hachinski V, Whitehead SN. Amyloid burden, neuroinflammation, and links to cognitive decline after ischemic stroke. Stroke. 2014; 45:2825–9. 10.1161/STROKEAHA.114.004285 25005439

[29] Bui TA, Jickling GC, Winship IR. Neutrophil dynamics and inflammaging in acute ischemic stroke: A transcriptomic review. Front Aging Neurosci. 2022; 14:1041333. 10.3389/fnagi.2022.1041333 36620775 PMC9813499

[30] Sharma S, Tyagi T, Antoniak S. Platelet in thrombo-inflammation: Unraveling new therapeutic targets. Front Immunol. 2022; 13:1039843. 10.3389/fimmu.2022.1039843 36451834 PMC9702553

[31] Denes A, Thornton P, Rothwell NJ, Allan SM. Inflammation and brain injury: acute cerebral ischaemia, peripheral and central inflammation. Brain Behav Immun. 2010; 24:708–23. 10.1016/j.bbi.2009.09.010 19770034

[32] Sun JH, Tan L, Yu JT. Post-stroke cognitive impairment: epidemiology, mechanisms and management. Ann Transl Med. 2014; 2:80. 10.3978/j.issn.2305-5839.2014.08.05 25333055 PMC4200648

[33] Lai KS, Liu CS, Rau A, Lanctôt KL, Köhler CA, Pakosh M, Carvalho AF, Herrmann N. Peripheral inflammatory markers in Alzheimer’s disease: a systematic review and meta-analysis of 175 studies. J Neurol Neurosurg Psychiatry. 2017; 88:876–82. 10.1136/jnnp-2017-316201 28794151

[34] Goulay R, Mena Romo L, Hol EM, Dijkhuizen RM. From Stroke to Dementia: a Comprehensive Review Exposing Tight Interactions Between Stroke and Amyloid-β Formation. Transl Stroke Res. 2020; 11:601–14. 10.1007/s12975-019-00755-2 31776837 PMC7340665

[35] Cheng Y, Zhu H, Sun D, Li L, Liu C, Sun S, Guo Y, Gu C, Gao Y, He G, Xue S. High triglyceride-glucose index at admission is a predictor of post-stroke cognitive impairment in patients with acute ischemic stroke. J Stroke Cerebrovasc Dis. 2024; 33:107510. 10.1016/j.jstrokecerebrovasdis.2023.107510 38000109

[36] Cheng Y, Zhu H, Chen J, Li L, Liu C, Gao Y, Sun D. Serum TG/HDL-C level at the acute phase of ischemic stroke is associated with post-stroke cognitive impairment. Neurol Sci. 2022; 43:5977–84. 10.1007/s10072-022-06267-6 35829832

[37] Nasreddine ZS, Phillips NA, Bédirian V, Charbonneau S, Whitehead V, Collin I, Cummings JL, Chertkow H. The Montreal Cognitive Assessment, MoCA: a brief screening tool for mild cognitive impairment. J Am Geriatr Soc. 2005; 53:695–9. 10.1111/j.1532-5415.2005.53221.x 15817019

